# Next-Generation Invaders? Hotspots for Naturalised Sleeper Weeds in Australia under Future Climates

**DOI:** 10.1371/journal.pone.0084222

**Published:** 2013-12-26

**Authors:** Daisy Englert Duursma, Rachael V. Gallagher, Erin Roger, Lesley Hughes, Paul O. Downey, Michelle R. Leishman

**Affiliations:** 1 Department of Biological Sciences, Macquarie University, Macquarie Park, Australia; 2 Institute for Applied Ecology, University of Canberra, Canberra, Australia; University of New South Wales, Australia

## Abstract

Naturalised, but not yet invasive plants, pose a nascent threat to biodiversity. As climate regimes continue to change, it is likely that a new suite of invaders will emerge from the established pool of naturalised plants. Pre-emptive management of locations that may be most suitable for a large number of potentially invasive plants will help to target monitoring, and is vital for effective control. We used species distribution models (SDM) and invasion-hotspot analysis to determine where in Australia suitable habitat may occur for 292 naturalised plants. SDMs were built in MaxEnt using both climate and soil variables for current baseline conditions. Modelled relationships were projected onto two Representative Concentration Pathways for future climates (RCP 4.5 and 8.5), based on seven global climate models, for two time periods (2035, 2065). Model outputs for each of the 292 species were then aggregated into single ‘hotspot’ maps at two scales: continental, and for each of Australia’s 37 ecoregions. Across Australia, areas in the south-east and south-west corners of the continent were identified as potential hotspots for naturalised plants under current and future climates. These regions provided suitable habitat for 288 and 239 species respectively under baseline climates. The areal extent of the continental hotspot was projected to decrease by 8.8% under climates for 2035, and by a further 5.2% by 2065. A similar pattern of hotspot contraction under future climates was seen for the majority of ecoregions examined. However, two ecoregions - Tasmanian temperate forests and Australian Alps montane grasslands - showed increases in the areal extent of hotspots of >45% under climate scenarios for 2065. The alpine ecoregion also had an increase in the number of naturalised plant species with abiotically suitable habitat under future climate scenarios, indicating that this area may be particularly vulnerable to future incursions by naturalised plants.

## Introduction

Over the last two decades, the potential for anthropogenic climate change to affect the distribution, physiology and management of invasive species has emerged as a major area for ecological research and policy development [1-3]. Given the significant economic burden and environmental consequences of invasive species [4], this push to understand how changing climate regimes may alter the dynamics of invasions is unsurprising. However, in the rush to understand how well-established invaders may respond, few studies have focused on the potential for changing climates to facilitate new invasions and little attention has been paid to the large pool of naturalised species from which the next generation of invaders may emerge. Pre-empting how naturalised species may respond to climate change and identifying locations in the landscape that may be most vulnerable to new invasions is an urgent goal for alien species management. 

Naturalised species are introduced, non-native organisms that have formed self-sustaining populations which are yet to significantly spread through the landscape or become invasive [5]. Progression through the three states of invasion (introduced, naturalised, and invasive) is conceptualised as a linear process where species pass through a series of abiotic and biotic barriers. For example, in plants, the transition from introduced to naturalised may involve overcoming barriers to reproduction (e.g. attracting mutualists for pollination) [6,7] and to establishment in different climates. Dubbed the ‘invasion-continuum’, this model of plant invasions has become a unifying tool for researchers over the last decade [5]. In a recent review [8], it was noted that much research has focused on the final transition of the invasion continuum, when species have already become widespread and have discernible impacts on native communities. However, as abiotic conditions continue to change under human influences, it is increasingly likely that a new suite of plant invaders will emerge; these species are likely to reside within the current pool of naturalised plants i.e. at the beginning of the invasion continuum [9,10]. Therefore, there is an urgent need to better predict locations susceptible to establishment of naturalised plants, in order to target emerging weed threats under changing climates. By identifying areas of the landscape that are at greatest risk from future invasions by introduced plants, those charged with managing invaders can make advance plans for maximising the effectiveness of control strategies.

Identifying the factors that drive invasiveness is a central goal for invasion science. Recent research has revealed general characteristics of invasive species that can be used to manage these species [11,12]. These include a range of factors that influence the transition from naturalised to invasive. Here we focus on climatic suitability which is a key determinant of a species’ range [13]. Consequently, the use of species distribution models (SDMs) calibrated with climate and environmental variables may be a particularly useful tool for identifying potential future invaders from the pool of naturalised species. In addition, models of species ranges can be aggregated into maps that provide area-based assessments of which ecosystems may be most vulnerable to future invasions. This type of analysis can be invaluable for developing pro-active landscape-scale management strategies and locating target regions for monitoring and eradication of alien species.

In Australia, almost 30,000 non-native plant species have been introduced since European settlement in the late 1700’s and around 10% of these introduced species are recognised as naturalised, but not yet invasive [14]. These naturalised plants, which are also referred to as ‘sleeper weeds’, pose a latent threat to native biodiversity [15,16]. In a global context, Australia has the highest species richness of naturalised plants of any biogeographic region [8]. This pool of naturalised plants is taxonomically distinct from the Australian flora (i.e. 26% of naturalised plants come from plant families not native to Australia based on comparative data from the Australian Plant Census (http://www.anbg.gov.au/chah/apc/); and their establishment has therefore led to the increasing homogenisation of Australia’s unique flora with other regions of the world. 

In this study, we assessed the potential for climate change to affect the distribution of naturalised, but not yet invasive, plants in Australia and used this information to identify hotspots of potentially suitable habitat (i.e. areas of habitat containing suitable climate and soils) for multiple naturalised species under current and future climate conditions. We used a combination of species distribution modelling and spatial analysis techniques to assess the potential effects of climate change on approximately 10% of Australia’s naturalised plants (*n* = 292 species). Our approach was to: (1) build models of abiotic suitability (based on climate variables and soil characteristics for observations within both the native and exotic ranges) under baseline climate conditions for the period 1950-2000, (2) project these models onto potential future climates for the decades centred around 2035 and 2065 to identify emergent patterns of potential expansion or contraction, and (3) use the concept of hotspot analysis for alien species (i.e. identifying key areas of high suitability for large numbers of non-native species [17]) to highlight the ecoregions within Australia that will provide the most suitable abiotic habitat for naturalised plants under current and future climates.

## Materials and Methods

### CRITERIA FOR SELECTING SPECIES

We focused on approximately 10% (*n* = 292 species) of the introduced plant species documented as naturalised, but not yet invasive, in Australia as the basis of this study [14] (see Table S1 for full list of species examined). Randall’s [14] checklist is the most comprehensive compilation of introduced plants in Australia and uses references in published literature to classify species into naturalised and invasive categories. We acknowledge that there may be some species included in our study for which individual populations may exhibit invasive behaviour in some habitats. However, the Randall checklist is the only national level inventory with standard criteria for separating introduced species into naturalised and invasive categories and is considered an authoritative source of information on invasion stage for introduced species [18].

Five criteria were used to select the 292 plant species from the checklist: (1) the species were listed as naturalised, but not invasive in [14], (2) the species were primarily terrestrial, and not reliant on the presence of permanent water for growth and reproduction (i.e. not aquatic species), (3) the species were not listed as noxious weeds (i.e. under legislation and therefore active control) in any state of Australia (www.weeds.org.au/noxious.htm), (4) the species were not native anywhere in Australia, and (5) more than 100 geo-referenced records were available for each species before data cleaning (after data cleaning procedures ~93% of species had >100 records). Application of this five criteria rule-set yielded a shortlist of ~1300 naturalised plants in Australia. The list was further refined, based on the availability of information on habitat and functional traits, resulting in a final list of 292 species that encompassed a range of functional types (e.g. vines, trees, shrubs, grasses, herbs; Table S1) so as to not bias the analysis towards any particular group of emerging invaders. 

Species distribution records for all 292 species were downloaded from Australia’s Virtual Herbarium (AVH; http://avh.ala.org.au/search) and the Global Biodiversity Information Facility (GBIF; http://data.gbif.org/occurrences/) in June 2012. Georeferenced herbarium specimens provide an important record of the distribution of plant species, and their use in niche modelling studies is widespread. However, it is important to note that herbarium records provide ‘presence-only’ data and therefore do not systematically capture areas where the species may actually be absent in the environmental space being modelled. Introduced species may be either ignored or targeted by collectors in the field, which may influence the representativeness of sampling [19]. Further, herbarium records should not be used without applying procedures to clean the data to remove errors arising from incorrect geocoding, and from specimens that are cultivated in gardens or agricultural trials. The cleaning procedures used to refine the ~3.5 million records obtained for this study are outlined in Appendix S1. Briefly, we removed all cultivated specimens, added georeferencing to observations that had detailed locations (but no latitude and longitude), removed duplicate observations within an equal-area 8 km grid cell, and removed the bias in sample density in Western Europe and the United States of America. This process resulted in 353,698 records for the 292 species, ranging from 36 records for *Hemizonia pungens* to 8,065 records for *Poa pratensis*, with an average of 1,211 records per species. These records were for both the native and non-native ranges combined. Within Australia the average number of observations across all 292 species was 126, with a range of 1-1581 records. 

### CLIMATE AND SOIL DATA

A combination of five bioclimatic variables and one soil characteristic was used to build models of suitable abiotic habitat for each of the 292 naturalised plant species. The climate variables chosen encompass both mean and extreme temperature and precipitation conditions across species’ ranges and were derived from raw data (maximum temperature, minimum temperature, precipitation) provided on the Worldclim website (www.worldclim.org) for the baseline period of 1950-2000, at a 5 arc-minute resolution. Hereafter, this baseline period is referred to as ‘current’ climate. The five bioclimatic variables used were mean annual temperature (MAT; °C), maximum temperature in the warmest month (MTWM; °C), minimum temperature in the coldest month (MTCM; °C), annual precipitation (AP; mm), and precipitation seasonality (PS; co-efficient of variation of AP).

In addition to the climate variables we also included topsoil clay fraction (TCF; % weight). Gridded data on TCF were derived from the Harmonized World Soil Database (HWSD; version 1.2; available at http://webarchive.iiasa.ac.at/Research/LUC/External-World-soil-database/HTML/). We assume that this soil factor will remain largely unchanged under future climate scenarios. Particle size determines soil texture and character, with soils that have a high proportion of clay particles able to retain water and minerals [20]. Thus TCF provides a reasonable approximation of soil and nutrient availability to plants, particularly in Australia which has a high proportion of highly-leached nutrient-poor soils. 

We sourced coarse resolution (0.5 x 0.5 degree or ~50 km x 50 km) future climate projections from http://climascope.tyndall.ac.uk for the time periods 2035 and 2065. We used seven global climate models (GCMs): MRI-CGCM232A, ukmo-hadgem1, mpi-echam5, gfdl-cm20, ukmo-hadcm3, csiro-mk30, ccsr-miroc32med (see Table S2 for summary details of the seven GCMs used) for decades centred on 2035 and 2065 and Representative Concentration Pathways (RCPs) 4.5 (CO_2_ stabilises at ~650 ppm by 2100) and 8.5 (CO_2_ rising to ~1370 ppm by 2100), which are low and high emission scenarios, respectively (see Appendix S2). 

Recent assessments of modelled precipitation for Australia found that the seven GCMs used are more effective, when used in combination, at simulating seasonal precipitation in Australia than the remaining 11 GCMs used in the Intergovernmental Panel on Climate Change (IPCC) Fourth Assessment Report [21,22]. The use of multiple GCMs helps to represent the uncertainties inherent in predicting future climate scenarios [23]. The seven models were chosen based on sensitivity tests for the optimal number of GCMs to be used in ensemble forecasting. The RCP data were downscaled to 5 arc-minutes using a cubic spline of the anomalies (deviance from modelled current and modelled future) for use with the GCMs. The WorldClim baseline or current climate was used to create the future climate scenarios using the methods outlined in [24]. All downscaling and bioclimatic variable generation was performed using the ‘climates’ package in R [25,26]. 

### MODEL CALIBRATION

The algorithm MaxEnt (version 3.3.3k) [27], driven by the dismo 0.7-23 package in R x64 v. 2.15.2, was used to build models of abiotically suitable habitat for the 292 naturalised plant species. MaxEnt has been used extensively to model species’ ranges using presence-only data and has consistently emerged as a well-performing approach for this task in comparative studies [28,29]. MaxEnt is based on the maximum entropy principle and yields a continuous probability index of environmental suitability across a spatial surface. A detailed description of MaxEnt can be found in Elith et al. [30] and Merrow et al. [31].

To calibrate MaxEnt, we explored the effect of using all combinations of: (1) all features, hinge and no threshold, no hinge and no threshold, (2) regulation multipliers of 1, 1.5 and 2, and (3) two sets of environmental variables, which included or excluded the soil variable (TCF) on model performance. We used five-fold cross validation partitions of the available data to limit spatial bias in the data used to train and test the models and assigned a random seed for each model run. All other MaxEnt settings were set to the default options identified in [28,32]. Model settings and environmental variables were assessed using Bayesian Information Criterion (BIC) and Akaike information criteria (AIC_c_) calculated in ENMTools in combination with expert opinion to choose the most parsimonious set of environmental variables [33-35]. Following these assessments, the final MaxEnt model settings were: no hinge, no threshold, regulation multiplier of 1, and the environmental variables: MAT, MTWM, MTCM, AP, PS, and TCF.

MaxEnt requires information from a selection of background points in order to build models of environmental suitability. Therefore, for each naturalised species we used the function Random Points in R package dismo [36] to generate 10,000 random background points. These points were restricted to areas within the same Köppen-Geiger climate classification zones (www.koeppen-geiger.vu-wien.ac.at) as occurrence records [37]. Multiple techniques for limiting background point selection have recently been assessed [38,39] and our method was chosen for its robust performance across all species in preliminary comparative exercises. In our study, for each species, any grid cell could have only one background point, and background points could not occur in the same cells as presence points. It is important to note that background points do not assume presence or absence and instead encompass the whole environmental range of a species [30].

### ASSESSING MODEL ACCURACY AND THRESHOLDING PREDICTIONS

We used two statistics to assess MaxEnt model accuracy: (1) area under the Receiver Operating Characteristic (ROC) curve (AUC) [40], and; (2) the threshold-dependent binomial test of omission. The AUC is a threshold-independent measure that assesses the rate of correct classification of presence points by the modelled function. In circumstances where background points are used to replace known absences for a species the AUC can approach, but not reach a value of 1. An AUC score>0.75 is considered to provide a useful level of discrimination [28]. 

AUC scores have a number of known limitations for assessing model accuracy, such as equally weighting rates of false-presence and false-absences and being sensitive to the spatial extent of the background data selected [38,41]. Therefore, we employed a second statistic (threshold-dependent binomial test of omission) to validate model accuracy. This test, also known as ‘fixed sensitivity’ [42] is based on thresholded model output and calculates the fraction of known presences that were predicted absent and assesses whether the omission rate is lower than that of a random prediction. To assess if our models outperformed random models we used an AUC score of >0.75 and a 90% fixed sensitivity binomial test of omission rate of <0.5. All species met these requirement, and for species that had <100 georeferenced observations the average AUC score was 0.94, with the lowest being 0.82, and the average binomial test of omission rate was ~0.006 with the largest being 0.05. Based on the test scores and a subjective visual inspection, we included these species in the analysis. 

Gridded maps of the seven GCM projections were averaged to produce a consensus forecast of abiotically suitable habitat across all species for each time-step (2035 and 2065) and RCP. The continuous data were converted to binary maps to depict areas of abiotically suitable and unsuitable habitat for each species using a threshold at which a fixed sensitivity of 90% was reached, meaning that 90% of the observations were included in the suitable habitat while the area of suitable habitat was minimised (also called a 10% omission rate, see 43 for a full discussion of thresholds for presence-only species distribution modelling).

We assessed the amount of suitable habitat in 2035 and 2065 using full-dispersal and no-dispersal scenarios. The full-dispersal scenario assumed that species can readily occupy any new area that is suitable while the no-dispersal scenario is the intersection of the currently suitably habitat with future suitable habitat and assumes that species are unable to move to a new area. Under RCP 8.5 in 2035 only 36 species had a decrease in range size of >5% under no-dispersal when compared with full dispersal, and of these species, only six had a decrease in the area of suitable habitat of >20%. For the 2065 projection, 53 species had a decrease in suitability of> 5% and 10 of these had a decrease> 20%. Given that 110 of these species are readily available for sale in Australia [14], human-mediated dispersal is highly likely, combined the limited number of species with a large change in the area of suitable habitat under the no-dispersal scenario, we chose to only use the full-dispersal scenario.

### INTERPRETING MODEL OUTPUTS: LIMITATIONS AND CAVEATS

The calibration of models for naturalised species poses a distinct set of challenges (see 39 for a thorough exploration of this topic). However, by deliberately and carefully controlling how MaxEnt models are fitted under situations where species may not be at equilibrium with climate (e.g. naturalised species which are yet to spread to all suitable regions within a novel range), the reliability of range predictions can be substantially increased [39]. We have addressed the issue of non-equilibrium when modelling the naturalised plants in this study in three ways. Firstly, models were calibrated using data from both the native range (where species are likely to be at equilibrium with environmental conditions) and from the non-native range, both in Australia and other regions of the world. This step has been shown to improve the ability to accurately capture the fundamental niche of species [44]. Secondly, we confirmed that the current Australian climate conditions under which each species occurs fell within the two-dimensional climate niche space defined by Annual Mean Temperature (AMT) and Annual Precipitation (AP) from all records outside Australia. That is, we assessed whether the global niche of the species would adequately capture the climate conditions under which Australian populations occur prior to modelling and found no evidence of climate niche shifts, where species could be shown to be occupying novel environments within Australia [45]. Finally, we reduced the complexity of model fitting procedures in MaxEnt (i.e. only linear, quadratic and product features were calculated) leading to smoother response curves where outlying records had less influence on the modelled distribution. 

### IDENTIFYING HOTSPOTS OF POTENTIALLY SUITABLE ABIOTIC HABITAT AT TWO SCALES

The concept of hotspot analysis for invasive plants [17] was used here to identify areas of high habitat suitability for all 292 naturalised plant species at two distinct scales: (1) continental (across all of Australia), and (2) within each of the 37 terrestrial ecoregions present in Australia (listed in Table 1). Ecoregions are spatially distinct zones in the landscape that contain well-defined assemblages of natural communities and species, such as Eastern Australia Mulga Shrublands [46]. Our aim in performing hotspot analysis at two scales was to provide both a national level assessment of vulnerability to naturalised plants under current and future climates, as well as a habitat specific estimate that recognises ecoregions as an important unit for conservation planning. 

**Table 1:** Summary statistics for continental and ecoregion level hotspots of potentially suitable habitat for 292 naturalised plants in Australia.

**REGION**	**CURRENT**			**RCP 4.5 2035**			**RCP 4.5 2065**			**RCP 8.5 2035**			**RCP 8.5 2065**		
	mean	SD	area km^2^	mean	SD	Δ area (%)	mean	SD	Δ area (%)	mean	SD	Δ area (%)	mean	SD	Δ area (%)
**Australia**	61.8	52.1	1784652	59.4	51.6	-5.7	57.9	51.5	-8.8	58.9	51.5	-6.8	55.4	51.2	-14
**Ecoregions**															
Arnhem Land tropical savannah	45.8	6.9	27232	45.7	6.6	-14.5	45.2	6.6	-28.7	45.5	6.6	-19.7	44.5	6.7	-46.9
Australian Alps montane grasslands	150.4	22.8	2815	153.3	21.4	9.8	155.5	20.7	19.6	154	21.1	12.2	158.1	19.9	46.4
Brigalow tropical savannah	75.3	12.4	84988	69.5	13.5	-31.9	66.9	13.8	-48.9	68.6	13.6	-37.5	62.5	13.9	-73.8
Cape York Peninsula tropical savannah	55.5	8.3	27265	55.3	8.2	-3.4	54.7	8.3	-18.1	55.1	8.3	-9.5	53.8	8.4	-34.1
Carnarvon xeric shrublands	19.8	9.6	21711	19.4	9.1	-3.5	18.5	8.8	-13.2	19.1	9	-6	17.1	8.3	-27.5
Carpentaria tropical savannah	31.3	6.2	81056	31.2	5.9	-7.6	30.8	5.9	-14.9	31.1	5.9	-9.6	30.2	6	-25.1
Central Ranges xeric scrub	34.7	8.5	57843	30.6	8.4	-49.4	28.6	8.1	-65.2	30	8.3	-55.1	25.2	7.3	-92.1
Coolgardie woodlands	107.2	30.7	33989	102.4	31.9	-5.6	99.6	32.2	-13.3	101.6	32	-7.1	95.3	32.8	-26.8
Eastern Australia mulga shrublands	52.7	10.9	58880	46.4	12.9	-45.7	43.5	13.1	-71.7	45.4	13	-53.9	39	13.1	-97.5
Eastern Australian temperate forests	127.7	27	53797	121.2	24.1	-33.6	119.5	23.5	-41.2	120.7	23.9	-35.7	116.5	22.7	-57
Einasleigh upland savannah	58	18.9	31004	56.6	17.9	-9.7	55.3	17.7	-15.7	56.2	17.8	-11.5	53.3	17.3	-26.5
Esperance mallee	153.4	24.7	26652	149.8	25.1	-37.9	147.9	25.3	-53.5	149.2	25.1	-44.9	145.1	25.6	-65.3
Eyre and York mallee	153.1	18	14354	153	17.9	-9	152.1	18.1	-12.5	152.7	17.9	-11	150.4	18.4	-20.9
Gibson desert	19.3	6.5	37643	17.7	6.8	-13.7	16.6	6.4	-31.6	17.4	6.7	-17.4	14.7	5.4	-75.5
Great Sandy-Tanami desert	18	8.4	168269	17.9	7.5	0.7	17.1	7	-11.2	17.7	7.4	-2.5	15.9	6.3	-29.8
Great Victoria desert	52.1	22	99754	49	22.6	-14.9	46.1	22.2	-28.3	48.1	22.5	-21.3	41.5	21.8	-40.7
Jarrah-Karri forest and shrublands	187.6	9.7	2050	186.5	8.2	-44.9	185	7.3	-79.4	186	7.9	-62.1	183.7	6.6	-96.6
Kimberly tropical savannah	35.2	8.6	76197	35.4	7.9	-4.7	35.1	7.7	-11.2	35.3	7.9	-7.3	34.7	7.5	-19.9
Mitchell grass downs	31	11.6	114126	28.7	9.5	-26.8	27.1	8.9	-42.3	28.1	9.3	-32.7	24.9	8.1	-63.2
Mount Lofty woodlands	174.2	29.1	5593	177.2	28.4	6.3	175.8	29.1	2.5	176.8	28.6	7.5	173.3	30.1	-6.3
Murray-Darling woodlands and mallee	121.2	29.2	48170	119.7	30.9	2.8	117.5	31.5	-1.5	119	31.1	1.2	113.2	32.8	-9.5
Naracoorte woodlands	198.1	24.3	6795	198.1	24.4	0	196.8	24.4	-2	197.8	24.4	0	194.4	24.4	-14
Nullarbor Plains xeric shrublands	108.2	25.8	47772	106.3	25.8	-12.6	104.2	26.3	-23.8	105.7	26	-15.5	100.5	27.2	-40.9
Pilbara shrublands	15.2	5.7	42332	16	6.2	16.3	15.4	6.1	1.8	15.8	6.2	12.1	14.7	6.1	-16.8
Queensland tropical rain forests	85.7	13	6990	81.3	11.5	-40.4	80.2	10.7	-49.6	81	11.2	-40.4	78.4	9.6	-74.9
Simpson desert	26.4	13	130488	22.5	11.2	-38.5	20.6	10.8	-52.7	21.9	11.1	-42.8	18.4	10.2	-69.8
Southeast Australia temperate forests	189.6	34.2	65790	187.3	36.2	0.8	186.3	37.1	0.4	187	36.5	0.4	184.1	39	1.1
Southeast Australia temperate savannah	97.3	29.9	78122	92.4	31.2	-22	90.2	31.3	-29.2	91.7	31.2	-23.9	86.4	31.2	-45
Southwest Australia savannah	74.9	29.7	39071	72.4	29.1	-21.3	70.5	29	-32.2	71.8	29.1	-25.6	67.7	28.7	-43.6
Southwest Australia woodlands	152.8	27.5	11133	149.3	27.5	-8.3	147.2	27.8	-10.2	148.7	27.6	-9	143.9	28.2	-19.8
Swan Coastal Plain Scrub and Woodlands	125	12.3	2532	123	12.5	-40.1	121.6	12.8	-51.6	122.6	12.6	-43.1	119	12.8	-63.2
Tasmanian Central Highland forests	182.7	42	4463	181.6	43.9	5.7	182.4	43.4	8.6	181.9	43.7	7.1	183.8	42.3	5.7
Tasmanian temperate forests	228.1	19.7	3395	227.6	20.4	9.3	228	20.3	30.3	227.7	20.4	13.1	228.7	19.9	45.5
Tasmanian temperate rain forests	188.2	37.3	7785	186.5	37.7	-3.4	188	37.2	-3.4	187	37.5	-1.7	190.5	36.2	4.8
Tirari-Sturt stony desert	64.7	32.2	90181	59.7	33.8	-8.5	57.2	33.2	-17.6	58.9	33.6	-11	53.4	32.4	-34.3
Victoria Plains tropical savannah	25.5	4.4	42461	26.5	4	29.8	26.3	4	16	26.5	4	23.4	26	4	-3.5
Western Australian Mulga shrublands	26.7	17.9	111271	26.6	15.8	-9.7	25.2	15.3	-21	26.2	15.6	-13.9	23	14.4	-34.7

The mean number of species per grid cell (and standard deviation) within the continental hotspot and for each of the 37 ecoregion level hotspots under current and future climate scenarios are shown. Changes in the areal extent of hotspots relative to current conditions under two future time periods (2035 and 2065) and two RCPs (4.5 and 8.5) are also provided.

**Figure 1:**
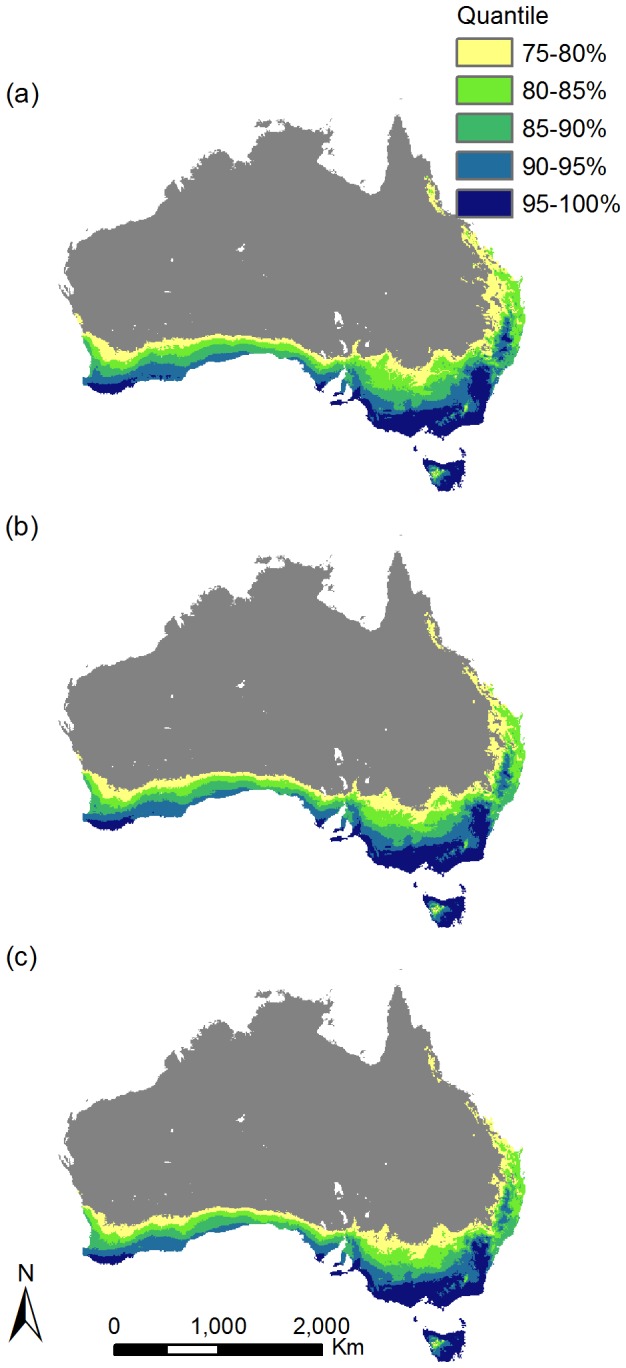
Maps of Australia showing the location of potential hotspots which represent the combined binary distributions of suitable habitat for 292 naturalised, but not yet invasive plant species based on the top 5^th^, 10^th^, 15^th^, 20^th^ and 25^th^ percentiles for: (a) current climate; (b) 2035 under RCP8.5, and; (c) 2065 under RCP8.5. The grey region represents areas where the combined suitability across all 292 species is less than the 25^th^ percentile.

Continental and ecoregion hotspots were calculated by summing the thresholded binary maps for each species and reclassifying grid cells with summed habitat suitability that was equal to, or above, the top 25th percentile of all grid cells. This is equivalent to identifying 25% of Australia, or 25% of an individual ecoregion, which is projected to provide suitable abiotic habitat for the most number of naturalised species. These reclassified grid cells were converted to binary hotspot maps (grid cells with values greater than or equal to the 25 percentile cut-off = 1, remaining grid cells = 0), using the *raster* package in R [48]. When visually mapping hotspots at the continental level, we used 5 percentile bands to show increasing habitat suitability and separate maps for each time-step (i.e. 2035, 2065). For ecoregions, we mapped the relative change in the areal extent of hotspots under future climates as a percentage of hotspot extent under current climate. That is, we applied the 25^th^ percentile cut-offs determined under current conditions to create hotspot maps for 2035 and 2065, enabling the potential hotspot contraction and/or expansion to be displayed. It is important to note that the statistics associated with hotspots are not directly comparable between ecoregions; they are deliberately intended to be habitat specific. Spatial information for the ecoregions was sourced from http://worldwildlife.org/publications/terrestrial-ecoregions-of-the-world (in March 2013) (Figure 1). All mapping was conducted using the ‘raster’ package version 2.0-21/r2529 in R [47].

## Results

### MODEL ACCURACY AND VARIABLE CONTRIBUTIONS

The average AUC score for the test data across the 292 naturalised species modelled in MaxEnt was 0.93 (±0.05), with AUC scores ranging between 0.78 and 0.99. These results indicate that for each species, the areas MaxEnt predicted to be abiotically suitable were correlated with the random 10% of observation data that was omitted from the species’ training records and was used to test the models. 

Averaged across all species, minimum temperature in the coldest month (MTCM) was the variable that contributed the most explanatory power (41.8%) for defining species’ distributions, with top soil clay fraction contributing the least (3.0%). The projections of suitable habitat indicated that 188 species will have a decrease in habitat suitability by 2065 while 65 will have little change and 39 will have an increase (see Table S1 for species level analysis of the area of abiotically suitable habitat under current , 2035 and 2065 conditions). 

### POTENTIAL HOTSPOTS OF SUITABLE ABIOTIC HABITAT UNDER CURRENT AND FUTURE CLIMATES

At a continental scale, we identified a band of suitable abiotic habitat for multiple species of naturalised plants under current conditions (a hotspot) that stretches from the Wet Tropics rainforest region in the northeast to south-west of Western Australia (Figure 1a). Of the 292 species modelled, 283 have at least one known population within this area, and this count does not change under future climate scenarios even though the hotspot contracts. Within this current hotspot, two areas, the south-west and the south-east of Australia (dark blue areas in Figure 1a), are of particular concern, as they provide suitable abiotic habitat for up to 288 of the 292 naturalised species examined (288 species in the south-east hotspot; 239 species in the south-west hotspot). Whilst the composition of species with suitable habitat did vary between these regions of extremely high suitability in the south-west and south-east of the continent, 239 species were common to both regions. The south-east hotspot had suitable abiotic habitat for 49 unique species not projected to occur in the south-west, including 15 grasses/sedges, 14 woody weeds, 13 herbaceous species and seven vine species. This compares with the south-west hotspot where there were no species projected to have suitable habitat that did not occur in the south-east hotspot. 

Across the continental hotspot, on average, 62 species (S.D. = +/-52 species) were projected to have suitable abiotic habitat in any single grid cell examined. However, some areas, such as the inland edges and tropical north-eastern portions of the hotspots, consistently had lower cumulative habitat suitability across species, suggesting that these areas may be less vulnerable to naturalised plant establishment and spread compared with more southerly coastal regions. Whilst our analysis provides composite hotspot maps, maps of suitable habitat for each of the 292 naturalised species are available at www.weedfutures.net.

For future climate scenarios, analyses were conducted for both RCP emission scenarios (4.5 and 8.5). Overall, there were similar patterns of variation between the two RCP scenarios, but RCP8.5 projected a larger decrease in hotspot area (Table 1). Presently, global CO_2_ emissions are tracking closer to RCP8.5 which is the ‘business as usual’ emissions pathway and appears likely to be the most realistic emission scenario unless strong mitigation is adopted [48]. For this reason, we present the results for the RCP 8.5 climate scenario for all analyses (however see Table 1 for data on RCP4.5).

Under future climate scenarios for both 2035 and 2065, the areal extent of the broad continental hotspot decreased in relation to that projected under current climate conditions (Figure 1b, c). In 2035, the hotspot reduced in area by 8.8% (157,050 sq km), with a further 5.2% (92,800 sq km) reduction projected for 2065. The majority of this change in areal extent was due to poleward shifts in projected species distributions, which resulted in fewer species having suitable habitat on the northern fringes of the hotspot in inland New South Wales and in tropical and subtropical Queensland. 

The mean number of naturalised plant species with suitable habitat in any given grid cell within the continental hotspot decreased from 62 under current conditions to 55 under scenarios for 2065 (Table 1). However, the maximum number of species in a single grid cell with suitable habitat under potential 2065 conditions remained stable (*n* = 249 species). The extent of suitable habitat was projected to decrease by>5% by 2065 for 50 species, and by >10% for 17 species when compared with the current hotspot patterns. These contractions were less pronounced for 2035, being 12 and zero species respectively. In total, 81.5% of species were projected to have at least some reduction in projected suitable habitat by 2065. However, 39 species showed expansions and 65 species had less than a 0.5% change in area of suitable habitat based on the percent of Australia that is currently suitable (see Table S1 for further details). 

**Figure 2:**
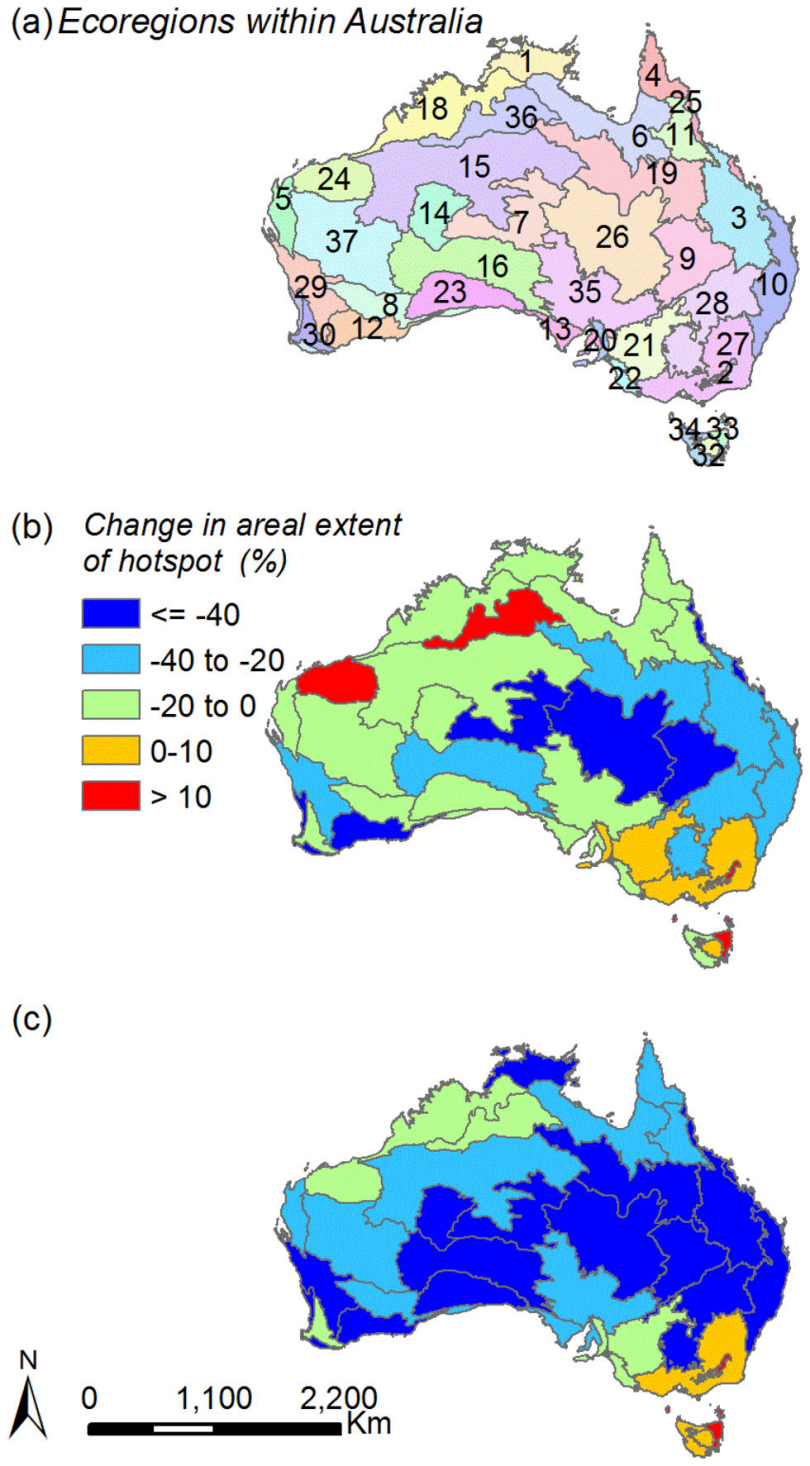
Changes in the areal extent of potential naturalised plant hotspots relative to current conditions within the 37 ecoregions of Australia. Maps depict: (a) the distribution of the 37 ecoregions of Australia (see below for ecoregion names), (b) the change in area of hotspot from current conditions to 2035 conditions in each ecoregion under RCP8.5, (c) as in (b) but for 2065 conditions. Ecoregion names: 1. Arnhem Land tropical savannah; 2. Australian Alps montane grasslands; 3. Brigalow tropical savannah; 4. Cape York Peninsula tropical savannah; 5. Carnarvon xeric shrublands; 6. Carpentaria tropical savannah; 7. Central Ranges xeric scrub; 8. Coolgardie woodlands; 9. Eastern Australia mulga shrublands; 10. Eastern Australian temperate forests, 11. Einasleigh upland savannah; 12. Esperance mallee; 13. Eyre and York mallee; 14. Gibson desert; 15. Great Sandy-Tanami desert; 16. Great Victoria desert; 17. Jarrah-Karri forest and shrublands; 18. Kimberly tropical savannah; 19. Mitchell grass downs; 20. Mount Lofty woodlands; 21. Murray-Darling woodlands and mallee; 22. Naracoorte woodlands; 23. Nullarbor Plains xeric shrublands; 24. Pilbara shrublands; 25. Queensland tropical rain forests; 26. Simpson desert; 27. Southeast Australia temperate forests; 28. Southeast Australia temperate savannah; 29. Southwest Australia savannah; 30. Southwest Australia woodlands; 31. Swan Coastal Plain scrub and woodlands; 32. Tasmanian Central Highland forests; 33. Tasmanian temperate forests; 34. Tasmanian temperate rain forests; 35. Tirari-Sturt stony desert; 36. Victoria Plains tropical savannah; 37. Western Australian Mulga shrublands.

At the ecoregion scale, we identified a similar pattern of hotspot contraction under future climate scenarios relative to current conditions as seen at the continental scale (Figure 2; Table 1). That is, the majority of the 37 ecoregions examined showed decreases in the areal extent of hotspots under future climates (2035: 29 ecoregions, Figure 2a; 2065: 32 ecoregions, Figure 2b). By 2065, the average decrease in hotspot area across all ecoregions was 44% and ranged from 97% in Eastern Australia mulga shrublands to 4% in Victoria Plains tropical savanna in the northwest of Australia (Table 1; Figure 2c). However in the remaining ecoregions, hotspots were projected to increase in area under RCP8.5 scenarios for both 2035 and 2065. For instance, in 2035, eight ecoregions had an increase in the area of their hotspots ranging from 0.4 to 23.4% (mean = 9.6%) and by 2065 five ecoregions had an increase in hotspot area ranging from 1.1% to 46.4% (mean = 20.7%). Of particular note was an increase in hotspot extent by >45% in Tasmanian temperate forests and Australian Alps montane grasslands under climate scenarios for 2065 (Table 1). 

On a per grid cell basis, most of the ecoregions are projected to experience a decrease in the mean number of naturalised plant species with suitable habitat by 2035 and 2065, relative to current conditions (Table 1). Interestingly, the particular ecoregions displaying these reductions were different between time steps. For instance, on average, potential conditions for 2035 saw a decrease in the mean number of species per grid cell in 32 ecoregions from 96 to 93 species. However, in 2065, whilst there were also 32 ecoregions with decreases in the mean number of species with suitable habitat per grid cell, the relative change was from 85 to 79 species. The pattern observed was due to continuing shifts in projections of suitable habitat between time-steps that differentially affect ecoregions, leading to different ecoregions showing increases in species composition with time. For example, by 2065, three of the five ecoregions experiencing increases in the mean number of species with suitable habitat were situated in Tasmania. This compares with 2035, where the majority of ecoregions with increasing numbers of species with suitable habitat were located on the mainland. 

Two ecoregions - Australian Alps montane grassland and Victoria Plains tropical savanna - had increasing numbers of species with suitable habitat in both 2035 and 2065. The Australian Alps montane grasslands had the greatest increase in the number of species with suitable habitat under future climate scenarios of any ecoregions examined. Of the 235 species with suitable habitat in the Australian Alps ecoregion, 135 species were projected to have an increase in suitable habitat, 89 species to have no change in suitable habitat (80 of these species with >99% of the region modelled as suitable habitat), and 21 species to experience a contraction in suitable habitat by 2065. 

## Discussion

As climatic conditions continue to change, it is increasingly likely that a new suite of plant invaders will emerge, many of which may already reside in the pool of naturalised species within a region. In this study we examined this emerging threat by identifying where potential hotspots of suitable habitat are located in the landscape for a representative sample of Australia’s naturalised, but not yet invasive, plant flora. At the continental scale, areas of extremely high suitability for a large proportion of naturalised plant species were identified in the south-west and south-east regions. However, the overall extent of the continental hotspot was projected to decline under future climate scenarios for 2035 and 2065. 

These findings are consistent with both O’Donnell et al. [17] and Gallagher et al. [37] who found that the extent of suitable habitat available for invasive species within the Australian continent was projected to decrease in the future, but that these areas still remained hotspots. In addition, there is a marked similarity in the location of the invasion-hotspots identified in this study and those identified by O’Donnell et al. [17] for 72 of the most serious invasive plants in Australia. Both studies highlight areas of south-west Western Australia and the south-east of the continent as containing suitable habitat for large numbers of non-native species. The current study, however, shows that a higher proportion of the land area of Tasmania is encompassed in the identified hotspot in all time-steps for naturalised but not yet invasive plants. The broad similarity in the location of hotspots between the two studies likely reflects both the favourable conditions available for plant growth in these temperate regions (as compared to the arid interior of the continent) and a tendency for both naturalised and invasive plants to be selected from the same species pool before introduction. That is, the majority of introduced plants in Australia have been imported for horticultural purposes [15] and this bias in introduction effort may skew the types of species in the non-native pool towards plants selected for growth in the relatively benign, temperate climate found in Australia’s south-east. In both hotspot studies, the proximity of these hotspot areas to Australia’s ‘intensive use zone’ (areas where extensive environmental modification and urbanisation has occurred), and the potential for high propagule pressure associated with nursery and garden supply of non-native plants in these areas is likely to increase future invasions [49]. Such a trend indicates the need for targeted monitoring in these areas under both current and future conditions to detect new naturalisations.

To date, most research has focused on how climate change could increase the success of those non-native species that are already established and widespread (i.e. invasive species) [e.g. 1, 3, 17, 37]. A number of potential responses of these plants to changing climate conditions have been documented, including projections of both decreasing [17,37] and increasing habitat suitability [50,51] for many species. Our results suggest that projections of future climate will alter the extent of suitable habitat for a large pool of naturalised species within Australia. We found that the size and intensity of potential hotspots for naturalised species will decrease at a continental scale under future climates. However, we also identified specific areas within Australia where invasion potential increased or remained high, suggesting that these areas could be vulnerable to new invasive species in the future. These areas span the south-east and south-west corners of Australia (Figure 1), with the south-east portion displaying the highest overall vulnerability. 

Similarly, hotspot areas calculated for individual ecoregions were largely projected to decline in extent under future climates. However, there were some notable exceptions where the potential hotspots were projected to expand by >45% of their current extent by 2065 (i.e. Australian Alps montane grasslands and Tasmanian temperate forest ecoregions). The Australian Alps montane grassland ecoregion also showed increases in the average number of species projected to have suitable habitat under future climates, making this ecoregion particularly vulnerable to naturalised plant establishment. Our ability to isolate individual ecoregions which may offer suitable abiotic habitat for the establishment of multiple naturalised plant species may inform current and future preventative management strategies.

The Australian Alps montane grasslands ecoregion includes the Australian alpine region that has been identified as an extremely vulnerable ecosystem to the effects of climate change [52]. The alpine zone has experienced decreases in snow cover since the 1960s [53]. Displacement of higher altitude species by lowland species shifting to higher elevations may have important consequences for native plant recruitment and community composition [54,55]. Similarly in Europe, native plant species from mountains have been shown to be disproportionately sensitive to climate change due to their narrow habitat tolerances [56]. This displacement of native species as a result of changing climate regimes in alpine areas may create additional recruitment opportunities for alien species [57]. Invasion of Australia’s alpine zone by non-native plants is increasing and our findings suggest that future climate conditions could place further pressure on this ecosystem. 

Previous studies have used the outputs of species distribution models to identify areas of particular conservation value/concern (e.g. for threatened plants, centres of endemism [58,59], weed invasion hotspots [17], and the relationship between native and alien diversity [60,61]). However, in order for hotspot analysis of any kind to be an effective tool for proactive conservation planning, analyses need to be performed at an appropriate scale [60]. For example, applying findings from hotspots derived at continental scales may be relatively uninformative for conservation planning or weed management in specific habitats. For this reason, we deliberately chose to calculate hotspots for individual ecoregions. In doing so, we were able to highlight how vulnerability to plant invasions varies across ecoregions and to provide guidance on which habitats may benefit from proactive monitoring of naturalised plants under changing climate regimes in coming decades. 

A recent review on the dynamics and distribution of naturalised plants [8] highlighted that to date, much research has focused on the final transition of the invasion continuum i.e. on those species that have become sufficiently widespread to have discernible impacts on native communities. Improving our understanding of how the likely next pool of invaders will respond to changing climate regimes is also a key concern for managers. Indeed, decision processes will be needed that enable policy makers and land managers to allocate priorities now to limit or prevent future invasions by naturalised species. For example, our research has identified invasion hotspots at an ecoregion scale; these should be priorities for general surveillance in regional early detection programs by land management agencies. Furthermore, any pathways between currently invaded and likely future invasive sites should be prioritised for monitoring and management.

It is important to recognise that many, if not all, of the species modelled in this study are yet to fully occupy their potential environmental niche as projected under baseline conditions. So, although climate models may predict that the amount of suitable habitat for some species may decrease in the future, such species still pose a threat in regions where climatic suitability remains stable between baseline and future conditions and where there are currently self-sustaining populations. Managing populations of naturalised species throughout these regions will be challenging, given both the potential scale of infestation and the need to coordinate responses among different stakeholders. In addition, the ability to transition from naturalised to invasive will not only be influenced by factors such as climatic and habitat suitability, but also by extreme events, propagule pressure, genetic drift and phenotypic variation. Deterministic and stochastic factors are also likely to influence the likelihood that naturalised plants become invaders [8]. For instance, the competitive and facilitative interactions between co-occurring plant species affect patterns of abundance and diversity. 

Naturalised plants may have established populations in sub-optimal parts of their range in Australia, as many have spread from gardens where supplementary water and nutrients are often provided. Where these species establish in areas with similar climates/soils to their native range, invasion may occur more rapidly. However, the ability of introduced species to shift climate niches in the non-native range should also be considered when assessing the probability of species becoming invasive [45]. It is also important to note that we have assessed a relatively small selection of the Australian naturalised plants, and although we found a general decline in the hotspots for naturalised plants there are more than 30,000 alien plants in Australia and future invasion rates may not change because a new cohort of invasive species may emerge.

### CONCLUSIONS

The central aim of this research was to assess the current extent of environmentally suitable habitat for a suite of naturalised, but not yet invasive non-native plants in Australia and to evaluate how projected changes in climate may alter these patterns in the coming decades. We found that changing climate regimes have the potential to create more favourable conditions for naturalised plants in some regions and thereby increase their invasive potential. However these findings varied greatly depending on the scale of analysis. Our findings highlight that assessment over multiple scales is critical because climate can influence species distributions along a continuum of spatial scales from biogeographic, landscape and microclimate patch-level effects [62,63]. As the gaps in our knowledge become clearer on a region-by-region basis, this information can be used by invasive plant management to prioritise control and eradication of those species expected to emerge as new threats or transformer species in the future.

## Supporting Information

Appendix S1 **The process used for cleaning the observational records prior to use in MaxEnt modelling.**
(DOCX)

Table S1 **The 292 naturalised, but not yet invasive plant species in Australia that were modelled in this study.**Accepted nomenclature follows the Plant List http://www.theplantlist.org/. % area of Australia providing potentially suitable abiotic habitat under current and future conditions for 2065 as modelled using MaxEnt are also provided.
(DOCX)

Table S2 **Global climate models used to simulate potential future climate scenarios across Australia.**
(DOCX)
