# Supplementary material for: Next-Generation Invaders? Hotspots for Naturalised Sleeper Weeds in Australia under Future Climates
Source: PLoS One. 2013 Dec 26;8(12):e84222. doi: 10.1371/journal.pone.0084222 (PMC3873406; doi:10.1371/journal.pone.0084222)
Supplement: Appendix S1 — The process used for cleaning the observational records prior to use in MaxEnt modelling. (DOCX) [file pone.0084222.s001.docx]

**APPENDIX S1**

**The process used for cleaning the observational records prior to use**

For non-Australian observations, we: (1) deleted records with no latitude and longitude coordinates, or those that had with low or poor spatial accuracy (i.e. within 10km; when accuracy was known), and; (2) deleted those records most likely to have been collected from cultivation by systematically searching the locality descriptions for the words ‘garden’, ‘nursery’, ‘greenhouse’, ‘ornamental’, ‘experimental’ and ‘cultivated’ in English, French, Dutch, Spanish, Portuguese, and Italian.

For Australian records, we (1) deleted records with no latitude and longitude coordinates – those that had a locality description, we used the Geoscience Australia Gazetteer of Australian Place Names Search (www.ga.gov.au/place-names/) and Google Earth (www.google.com/earth/index.html) to assign approximate coordinates, and; (2) manually processed the records containing the words ‘garden’, ‘nursery’, ‘greenhouse’, ‘ornamental’, ‘experimental’ and ‘cultivated’ to limit false removals (e.g. a weed growing next to garden, versus purposely planted as an ornamental in a private garden).

For all records we deleted duplicate records and limited records to one observation for a species per 8 km equal area grid cell, thereby defining a record as presence within a specific grid cell and hereafter referred to as gridded observations. In addition, for 139 species that had more than 200 gridded observations and 75% of the observations occurring in densely sampled regions within Europe and the USA, we further reduced the number of records in these areas so that one record for each species was permitted to occur in a 24 km equal area grid cell. This step reduced sampling bias in the areas where species have been extensively and systematically collected and recorded in GBIF, and the reduction in bias increased model performance when delineating suitable and non-suitable habitat (see *Assessing model accuracy and thresholding predictions*). By applying this procedure, we removed records from the densely sampled regions, resulting in an effective ‘up-weighting’ of records from outside these locations [36].

The final cleaned global dataset was then mapped using the Raster 2.0-21 package [43] in R x64 2.15.2 (Hijmans et al., 2012) and clipped to a mask of global land surface boundaries (land mask created from gridded climate and soil layers used in modelling).

**Justification of RCP use**

RCPs are a relatively new, standardised method for capturing potential warming trajectories which are based on amounts of atmospheric radiative forcing expected by the year 2100, measured in W/m^2^ (Moss et al. 2010; Van Vuuren et al. 2011). RCP8.5 represents a rising radiative forcing trajectory (i.e. greenhouse gas emissions rising to an equivalent of ~1370 ppm CO_2_ equivalent by 2100), whilst RCP4.5 is a more conservative pathway, estimating a stabilisation of radiative forcing by 2100 (~650 ppm CO_2_ equivalent after stabilisation at 2100). RCPs have been adopted by the IPCC to replace the Special Report on Emissions Scenarios (SRES) used in the AR4 report (Solomon et al. 2007); RCPs will be used in the AR5 IPCC report due to be published in 2014. Although new GCM runs for RCPs have not been fully completed, several research groups have implemented methods to utilise knowledge gained from SRES predictions to recreate predictions for the new RCPs using AR4 GCMs (e.g. [Meinshausen, Raper & Wigley 2011a; Meinshausen et al. 2011b; Rogelj, Meinshausen & Knutti 2012]). The methods used to generate the GCM predictions for the RCP emission scenarios are defined at [http://climascope.wwfus.org](http://climascope.wwfus.org/) and in associated publications (e.g. [Mitchell & Jones 2005, Warren et al. 2008, Meinshausen et al. 2011a]).

**Steps to improve model accuracy**

The calibration of models for naturalised plant species poses a distinct set of challenges (see Elith et al. 2010 for a thorough exploration of this topic). However, by deliberately and carefully controlling how MaxEnt models are fitted under situations where species may not be at equilibrium with climate (e.g. naturalised species which are yet to spread [invasive] to all suitable regions within a novel range), the reliability of range predictions can be substantially increased (Elith et al. 2010). We have addressed the issue of non-equilibrium when modelling the naturalised plants in this study in three ways. Firstly, models were calibrated using data from both the native range (where species are likely to be at equilibrium with environmental conditions) and from the entire naturalised range, both in Australia and other regions of the world. This step has been shown to improve accurate capture of the fundamental niche of species (Beaumont et al. 2009). Secondly, we confirmed that the current Australian climate conditions under which each species occurs fell within the two-dimensional climate niche space defined by Annual Mean Temperature (AMT) and Annual Precipitation (AP) from all records outside Australia. That is, we assessed whether the global niche of the species would adequately capture the conditions under which Australian populations occur prior to modelling and found no evidence of climate niche shifts, where species could be shown to be occupying novel environments within Australia (Gallagher *et al.* 2010). Finally, we reduced the complexity of model fitting procedures in MaxEnt (i.e. only linear, quadratic and product features were calculated) leading to smoother response curves where outlying records had less influence on the modelled distribution. Note that we also tested increasing the beta-multiplier from 1 to 2.5 (*sensu* Elith et al. 2010), but found this step had no significant effect on model predictions across all species. These measures decreased the likelihood that models captured the dynamics of non-equilibrium species.

**REFERENCES**

Beaumont LJ, Gallagher RV, Thuiller W, Downey PO, Leishman MR, et al. (2009) Different climatic envelopes among invasive populations may lead to underestimations of current and future biological invasions. Diversity and Distributions 15: 409-420.

Elith J, Kearney M, Phillips S (2010) The art of modelling range-shifting species. Methods in Ecology and Evolution 1: 330-342.

Gallagher RV, Beaumont LJ, Hughes L, Leishman MR (2010) Evidence for climatic niche and biome shifts between native and novel ranges in plant species introduced to Australia. Journal of Ecology 98: 790-799.

Hijmans RJ, Van etten J (2012) Raster: Geographic analysis and modeling with raster data. R package version 2.0-21/r2529 ed.

Meinshausen M, Raper SCB, Wigley TML (2011) Emulating coupled atmosphere-ocean and carbon cycle models with simpler model, MAGICC6-Part 1: Model description and calibration. Atmospheric Chemistry and Physics 11: 1417-1456.

Meinshausen M, Smith SJ, Calvin K, Daniel JS, Kainuma MLT, et al. (2011) The RCP greenhouse gas concentrations and their extensions from 1765 to 2300. Climatic Change 109: 213-241.

Mitchell TD, Jones PD (2005) An improved method of constructing a database of monthly climate observations and associated high-resolution grids. International Journal of Climatology 25: 693-712.

Moss RH, Edmonds JA, Hibbard KA, Manning MR, Rose SK, et al. (2010) The next generation of scenarios for climate change research and assessment. Nature 463: 747-756.

Rogelj J, Meinshausen M, Knutti R (2012) Global warming under old and new scenarios using IPCC climate sensitivity range estimates. Nature Climate Change 2: 248-253.

Solomon S, Quin D, Manning M, Chen Z, Marquis M, et al. (2007) Climate chnage 2007: Synthesis report. Contribution of working group I, II, and III to the fourth assessment report of the Intergovernmental Panel on Climate Change. Summary for Policymakers. Intergovernmental Panel on Climate Change (IPCC), Geneva.

Van Vuuren D, Edmonds J, Kainuma M, Riahi K, Thomson A, et al. (2011) The representative

concentration pathways: an overview. Climatic Change 109: 5-31.

Warren DE, Glor RE, Turelli M (2010) ENMTools: a toolbox for comparative studies of environmental niche models. Ecography 33: 607-611.
