# Supplementary material for: Next-Generation Invaders? Hotspots for Naturalised Sleeper Weeds in Australia under Future Climates
Source: PLoS One. 2013 Dec 26;8(12):e84222. doi: 10.1371/journal.pone.0084222 (PMC3873406; doi:10.1371/journal.pone.0084222)
Supplement: Table S1 — The 292 naturalised, but not yet invasive plant species in Australia that were modelled in this study.Accepted nomenclature follows the Plant List http://www.theplantlist.org/. % area of Australia providing potentially suitable abiotic habitat under current and future conditions for 2065 as modelled using MaxEnt are also provided. (DOCX) [file pone.0084222.s002.docx]

**TABLE S1:** The 292 naturalised, but not yet invasive plant species in Australia that were modelled in this study. Accepted nomenclature follows the Plant List <http://www.theplantlist.org/>. % area of Australia providing potentially suitable abiotic habitat under current and future conditions for 2065 as modelled using MaxEnt are also provided.

| **Accepted nomenclature** | **Growth form(s)** | **Family** | **Current** | **RCP8.5 2035** | **RCP 8.5 2065** |
| --- | --- | --- | --- | --- | --- |
| Acanthocereus tetragonus (L.) Hummelinck | Succulent | Cactaceae | 19 | 18 | 15 |
| Aeonium arboreum Webb & Berthel. | Succulent shrub | Crassulaceae | 17 | 16 | 16 |
| Aeschynomene americana L. | Herb, Shrub | Fabaceae | 14 | 14 | 14 |
| Agapanthus praecox Willd. | Herb | Agapanthaceae | 8 | 7 | 7 |
| Agrostis capillaris L. | Graminoid | Poaceae | 8 | 7 | 7 |
| Agrostis gigantea Roth | Graminoid | Poaceae | 6 | 6 | 5 |
| Agrostis stolonifera L. | Graminoid, Herb | Poaceae | 11 | 10 | 9 |
| Aira caryophyllea L. | Graminoid | Poaceae | 14 | 13 | 12 |
| Alopecurus aequalis Sobol. | Graminoid | Poaceae | 7 | 6 | 6 |
| Alopecurus geniculatus L. | Graminoid | Poaceae | 18 | 15 | 12 |
| Alopecurus myosuroides Huds. | Graminoid | Poaceae | 8 | 8 | 7 |
| Alopecurus pratensis L. | Graminoid | Poaceae | 5 | 4 | 4 |
| Annona squamosa L. | Tree | Annonaceae | 15 | 15 | 15 |
| Anthoxanthum aristatum Boiss. | Graminoid | Poaceae | 8 | 8 | 7 |
| Anthoxanthum odoratum L. | Graminoid | Poaceae | 9 | 8 | 7 |
| Anthriscus caucalis M.Bieb. | Herb | Apiaceae | 6 | 5 | 5 |
| Antigonon leptopus Hook. & Arn. | Vine/scrambler/climber | Polygonaceae | 55 | 54 | 52 |
| Arctium lappa L. | Herb | Asteraceae | 4 | 4 | 4 |
| Arrhenatherum elatius (L.) P.Beauv. ex J.Presl & C.Presl. | Graminoid | Poaceae | 7 | 6 | 6 |
| Arum italicum Mill. | Herb | Araceae | 9 | 9 | 8 |
| Atriplex hortensis L. | Herb | Chenopodiaceae | 6 | 5 | 5 |
| Avena fatua L. | Graminoid | Poaceae | 39 | 35 | 32 |
| Avena sativa L. | Graminoid | Poaceae | 31 | 28 | 26 |
| Avena sterilis L. | Graminoid | Poaceae | 29 | 29 | 29 |
| Berberis vulgaris L. | Shrub | Berberidaceae | 4 | 3 | 3 |
| Betula pendula Roth | Tree | Betulaceae | 4 | 3 | 3 |
| Bixa orellana L. | Shrub, Tree | Bixaceae | 4 | 5 | 5 |
| Bougainvillea glabra Choisy | Vine/scrambler/climber | Nyctaginaceae | 40 | 39 | 36 |
| Brachypodium distachyon (L.) P.Beauv. | Graminoid | Poaceae | 21 | 22 | 22 |
| Brassica oleracea L. | Herb | Brassicaceae | 20 | 19 | 18 |
| Brassica rapa L | Herb | Brassicaceae | 18 | 17 | 16 |
| Briza maxima L. | Graminoid | Poaceae | 22 | 23 | 23 |
| Briza minor L. | Graminoid | Poaceae | 30 | 28 | 27 |
| Bromus hordeaceus L. | Graminoid | Poaceae | 19 | 18 | 17 |
| Bromus inermis Leyss. | Graminoid | Poaceae | 6 | 5 | 5 |
| Bromus lanceolatus Roth | Graminoid | Poaceae | 12 | 13 | 14 |
| Bromus madritensis L. | Graminoid | Poaceae | 21 | 20 | 19 |
| Bromus racemosus L. | Graminoid | Poaceae | 5 | 4 | 4 |
| Bromus rubens L. | Graminoid | Poaceae | 29 | 27 | 25 |
| Bromus secalinus L. | Graminoid | Poaceae | 6 | 5 | 5 |
| Bromus tectorum L. | Graminoid | Poaceae | 8 | 7 | 7 |
| Buddleja davidii Franch. | Shrub | Scrophulariaceae | 6 | 6 | 5 |
| Caesalpinia gilliesii (Hook.) D.Dietr. | Shrub | Fabaceae | 54 | 52 | 50 |
| Cajanus cajan (L.) Millsp. | Shrub | Fabaceae | 21 | 23 | 24 |
| Calamagrostis epigeios (L.) | Graminoid | Poaceae | 4 | 3 | 3 |
| Calyptocarpus vialis Less. | Herb | Asteraceae | 56 | 54 | 48 |
| Calystegia silvatica (Kit. in Schrad.) Griseb. / Calystegia silvatica subsp. fraterniflora (Mack. & Bush) Brummitt | Vine/scrambler/climber | Convolvulaceae | 4 | 4 | 4 |
| Capsicum annuum L. | Shrub | Solanaceae | 44 | 42 | 41 |
| Cardamine corymbosa Hook.f. | Herb | Brassicaceae | 2 | 1 | 1 |
| Carex disticha Huds. | Graminoid | Cyperaceae | 2 | 2 | 2 |
| Carex divisa Huds. | Graminoid, Herb | Cyperaceae | 18 | 18 | 17 |
| Carex divulsa Stokes | Graminoid | Cyperaceae | 7 | 6 | 6 |
| Carex pilulifera L. | Graminoid | Cyperaceae | 3 | 3 | 3 |
| Carex punctata Gaudin | Graminoid | Cyperaceae | 4 | 4 | 3 |
| Carex scoparia Willd. | Graminoid | Cyperaceae | 1 | 1 | 1 |
| Casimiroa edulis La Llave | Tree | Rutaceae | 10 | 11 | 13 |
| Catapodium rigidum (L.) C.E.Hubb. | Graminoid | Poaceae | 16 | 16 | 15 |
| Centrosema pascuorum Benth. | Herb, Vine/scrambler/climber | Fabaceae | 12 | 12 | 12 |
| Centrosema plumieri (Pers.) Benth. | Vine/scrambler/climber | Fabaceae | 6 | 6 | 6 |
| Cerasus lusitanica (L.) Dum. Cours. | Shrub, Tree | Rosaceae | 4 | 3 | 3 |
| Ceratonia siliqua L. | Shrub, Tree | Fabaceae | 15 | 16 | 19 |
| Cestrum aurantiacum Lindl. | Shrub | Solanaceae | 9 | 9 | 8 |
| Cestrum elegans (Brongn. ex Neumann) Schltdl. | Shrub | Solanaceae | 8 | 8 | 7 |
| Cestrum nocturnum L. | Shrub | Solanaceae | 7 | 7 | 7 |
| Chloris pilosa Schumach. & Thonn. | Graminoid | Poaceae | 18 | 18 | 19 |
| Chloris virgata Sw. | Graminoid | Poaceae | 86 | 85 | 83 |
| Chorispora tenella (Pall.) DC. | Herb | Brassicaceae | 2 | 2 | 2 |
| Cichorium intybus L. | Herb | Asteraceae | 17 | 15 | 13 |
| Citrullus lanatus (Thunb.) Matsum. & Nakai | Herb, Vine/scrambler/climber | Cucurbitaceae | 97 | 96 | 96 |
| Clematis flammula L. | Vine/scrambler/climber | Ranunculaceae | 14 | 14 | 15 |
| Clitoria ternatea L. | Vine/scrambler/climber | Fabaceae | 29 | 28 | 27 |
| Cocos nucifera L. | Tree | Arecaceae | 4 | 4 | 4 |
| Codiaeum variegatum (L.) Rumph. ex A.Juss. | Shrub | Euphorbiaceae | 5 | 6 | 6 |
| Coix lacryma-jobi L. | Graminoid | Poaceae | 8 | 8 | 7 |
| Conyza primulifolia (Lam.) Cuatrec. & Lourteig | Herb | Asteraceae | 43 | 39 | 35 |
| Conyza sumatrensis (S.F.Blake) Pruski & G.Sancho | Herb | Asteraceae | 29 | 26 | 23 |
| Cordyline australis (G.Forst.) Endl. | Tree | Asparagaceae | 2 | 2 | 1 |
| Cotoneaster divaricatus Rehder & E.H. Wilson | Shrub | Rosaceae | 1 | 1 | 1 |
| Cotoneaster horizontalis Decne. | Shrub | Rosaceae | 3 | 3 | 3 |
| Crataegus pontica C. Koch | Shrub, Tree | Rosaceae | 20 | 21 | 21 |
| Crotalaria agatiflora Schweinf. | Shrub | Fabaceae | 10 | 10 | 10 |
| Crotalaria incana L. | Shrub | Fabaceae | 55 | 49 | 44 |
| Crotalaria pallida Aiton | Herb, Shrub | Fabaceae | 52 | 47 | 41 |
| Cucumis anguria L. | Herb | Cucurbitaceae | 60 | 60 | 59 |
| Cucumis dipsaceus Ehrenb. ex Spach | Herb, Vine/scrambler/climber | Cucurbitaceae | 53 | 46 | 41 |
| Cucumis myriocarpus Naudin | Herb | Cucurbitaceae | 54 | 52 | 48 |
| Cucurbita maxima Duchesne | Vine/scrambler/climber | Cucurbitaceae | 43 | 43 | 41 |
| Cupressus macrocarpa Hartw. | Tree | Cupressaceae | 4 | 4 | 4 |
| Cynara cardunculus L | Herb | Asteraceae | 25 | 25 | 25 |
| Cynosurus cristatus L. | Graminoid | Poaceae | 4 | 4 | 4 |
| Cynosurus echinatus L. | Graminoid, Herb | Poaceae | 12 | 12 | 12 |
| Cyperus aggregatus (Willd.) Endl. | Herb | Cyperaceae | 39 | 37 | 34 |
| Cyperus eragrostis Lam. | Graminoid, Herb | Cyperaceae | 31 | 29 | 26 |
| Dactylis glomerata L. | Graminoid | Poaceae | 17 | 16 | 15 |
| Dactyloctenium aegyptium (L.) Willd. | Graminoid | Poaceae | 74 | 70 | 63 |
| Desmanthus virgatus (L.) Willd. | Herb | Fabaceae | 70 | 66 | 59 |
| Desmodium intortum (Mill.) Urb. | Herb | Fabaceae | 10 | 10 | 10 |
| Desmodium tortuosum (Sw.) DC. | Herb, Shrub | Fabaceae | 45 | 40 | 32 |
| Dichanthium annulatum (Forssk.) Stapf | Graminoid | Poaceae | 77 | 78 | 80 |
| Dichrostachys cinerea (L.) Wight & Arn. | Shrub, Tree | Fabaceae | 47 | 48 | 48 |
| Digitaria ischaemum (Schreb.) Muhl. | Graminoid | Poaceae | 7 | 6 | 5 |
| Digitaria ternata (A.Rich.) Stapf | Graminoid | Poaceae | 17 | 17 | 17 |
| Digitaria violascens Link | Graminoid | Poaceae | 35 | 31 | 25 |
| Disa bracteata Sw. | Herb | Orchidaceae | 9 | 8 | 8 |
| Draba nemorosa L. | Herb | Brassicaceae | 1 | 1 | 1 |
| Ecballium elaterium (L.) A.Rich. | Herb | Cucurbitaceae | 19 | 18 | 17 |
| Echinochloa crus-pavonis (Kunth) Schult. | Graminoid, Herb | Poaceae | 56 | 55 | 54 |
| Echinochloa esculenta (A.Braun) H.Scholz | Graminoid | Poaceae | 29 | 24 | 20 |
| Echinochloa frumentacea Link | Graminoid, Herb | Poaceae | 40 | 35 | 30 |
| Echinochloa muricata (P.Beauv.) Fernald | Graminoid | Poaceae | 8 | 7 | 6 |
| Echinochloa oryzoides (Ard.) Fritsch | Graminoid | Poaceae | 40 | 38 | 36 |
| Ehrharta calycina Sm. | Graminoid, Herb | Poaceae | 23 | 23 | 22 |
| Eleusine tristachya (Lam.) Lam. | Graminoid | Poaceae | 27 | 24 | 21 |
| Elytrigia repens Desv. | Graminoid, Herb | Poaceae | 8 | 7 | 6 |
| Eragrostis atrovirens (Desf.) Trin. ex Steud. | Graminoid | Poaceae | 26 | 27 | 26 |
| Eragrostis bahiensis Roem. & Schult. | Graminoid, Herb | Poaceae | 57 | 51 | 43 |
| Eragrostis barrelieri Daveau | Graminoid, Herb | Poaceae | 68 | 67 | 64 |
| Eragrostis mexicana (Hornem.) Link | Graminoid, Herb | Poaceae | 31 | 35 | 37 |
| Eragrostis patula (Kunth) Steud. | Graminoid | Poaceae | 32 | 29 | 25 |
| Eragrostis pilosa (L.) P.Beauv. | Graminoid | Poaceae | 58 | 55 | 52 |
| Eragrostis tef (Zucc.) Trotter | Graminoid | Poaceae | 26 | 24 | 21 |
| Erica arborea L. | Shrub | Ericaceae | 11 | 11 | 11 |
| Eriobotrya japonica (Thunb.) Lindl. | Tree | Rosaceae | 28 | 26 | 24 |
| Eugenia uniflora L. | Shrub, Tree | Myrtaceae | 45 | 35 | 28 |
| Euphorbia cyparissias L. | Herb | Euphorbiaceae | 4 | 3 | 3 |
| Euphorbia helioscopia L. | Herb | Euphorbiaceae | 15 | 14 | 14 |
| Euphorbia paralias L. | Herb | Euphorbiaceae | 8 | 8 | 8 |
| Euphorbia platyphyllos L. | Herb | Euphorbiaceae | 4 | 4 | 4 |
| Fallopia convolvulus (L.) ç.Lšve | Herb | Polygonaceae | 14 | 13 | 11 |
| Festuca arundinacea Schreb. | Graminoid | Poaceae | 17 | 15 | 14 |
| Festuca pratensis Huds. | Graminoid | Poaceae | 5 | 5 | 4 |
| Ficus carica L. | Shrub, Tree | Moraceae | 23 | 23 | 24 |
| Florestina tripteris DC. | Herb | Asteraceae | 66 | 69 | 67 |
| Fraxinus excelsior L. | Tree | Oleaceae | 4 | 3 | 3 |
| Fumaria muralis Sond. ex W.D.J.Koch | Herb, Vine/scrambler/climber | Papaveraceae | 18 | 17 | 15 |
| Galium palustre L. | Herb | Rubiaceae | 4 | 4 | 4 |
| Gamochaeta americana (Mill.) Wedd. | Herb | Asteraceae | 17 | 16 | 14 |
| Gaura lindheimeri Engelm. & A.Gray | Herb | Onagraceae | 39 | 30 | 19 |
| Gazania linearis (Thunb.) Druce | Herb | Asteraceae | 27 | 25 | 23 |
| Gazania rigens (L.) Gaertn. | Herb | Asteraceae | 27 | 23 | 20 |
| Gladiolus communis L. | Herb | Iridaceae | 8 | 8 | 8 |
| Gossypium barbadense L. | Shrub | Malvaceae | 23 | 19 | 18 |
| Gossypium hirsutum L. | Herb, Shrub | Malvaceae | 63 | 56 | 50 |
| Gossypium thurberi (Unresolved) | Shrub, Tree | Malvaceae | 4 | 7 | 8 |
| Hebe elliptica (G.Forst.) Pennell | Shrub | Plantaginaceae | 0 | 0 | 0 |
| Hedysarum coronarium L. | Herb | Fabaceae | 13 | 15 | 17 |
| Heimia salicifolia (Kunth) Link | Shrub | Lythraceae | 31 | 33 | 33 |
| Hemizonia pungens (Hook. & Arn.) Torr. & A.Gray | Herb | Asteraceae | 22 | 22 | 23 |
| Holcus lanatus L. | Graminoid | Poaceae | 14 | 13 | 12 |
| Holcus mollis L. | Graminoid | Poaceae | 3 | 3 | 3 |
| Hordeum secalinum Schreb. | Graminoid | Poaceae | 3 | 2 | 2 |
| Hordeum vulgare L. | Graminoid | Poaceae | 34 | 34 | 34 |
| Humulus lupulus L. | Herb, Vine/scrambler/climber | Cannabaceae | 5 | 5 | 5 |
| Hylocereus undatus (Haw.) Britton & Rose | Succulent, Vine/scrambler/climber | Cactaceae | 26 | 25 | 23 |
| Hypericum humifusum L. | Herb | Hypericaceae | 4 | 4 | 3 |
| Hyptis spicigera Lam. | Shrub | Lamiaceae | 22 | 23 | 23 |
| Indigofera spicata Forssk. | Herb | Fabaceae | 16 | 15 | 14 |
| Indigofera tinctoria L. | Herb, Shrub | Fabaceae | 33 | 29 | 26 |
| Ipomoea aquatica Forssk. | Herb, Vine/scrambler/climber | Convolvulaceae | 34 | 35 | 36 |
| Ipomoea batatas (L.) Poir. | Vine/scrambler/climber | Convolvulaceae | 10 | 10 | 10 |
| Ipomoea carnea Jacq. | Shrub | Convolvulaceae | 34 | 26 | 19 |
| Ipomoea grandifolia (Dammer) O'Donell | Vine/scrambler/climber | Convolvulaceae | 25 | 25 | 24 |
| Ipomoea hederifolia L. | Vine/scrambler/climber | Convolvulaceae | 25 | 25 | 24 |
| Ipomoea mauritiana Jacq. | Herb | Convolvulaceae | 4 | 4 | 4 |
| Ipomoea nil (L.) Roth | Herb, Vine/scrambler/climber | Convolvulaceae | 70 | 67 | 60 |
| Ipomoea obscura (L.) Ker Gawl. | Vine/scrambler/climber | Convolvulaceae | 29 | 27 | 25 |
| Ipomoea pandurata (L.) G. Mey. | Vine/scrambler/climber | Convolvulaceae | 3 | 1 | 0 |
| Ipomoea quamoclit L. | Vine/scrambler/climber | Convolvulaceae | 39 | 32 | 24 |
| Iris foetidissima L. | Herb | Iridaceae | 3 | 3 | 3 |
| Isolepis marginata (Thunb.) A.Dietr. | Graminoid, Herb | Cyperaceae | 15 | 14 | 14 |
| Juncus acuminatus Michx. | Herb | Juncaceae | 7 | 7 | 6 |
| Juncus bulbosus L. | Herb | Juncaceae | 5 | 4 | 4 |
| Juncus canadensis J.Gay ex Laharpe | Graminoid | Juncaceae | 3 | 3 | 2 |
| Juncus capitatus Weigel | Graminoid, Herb | Juncaceae | 13 | 13 | 12 |
| Juncus conglomeratus L. | Graminoid | Juncaceae | 3 | 3 | 3 |
| Juncus fontanesii J.Gay ex Laharpe | Graminoid | Juncaceae | 20 | 20 | 20 |
| Juncus inflexus L. | Graminoid, Herb | Juncaceae | 8 | 7 | 7 |
| Juncus microcephalus Kunth | Herb | Juncaceae | 15 | 15 | 15 |
| Juncus tenuis Willd. | Graminoid | Juncaceae | 7 | 6 | 6 |
| Kniphofia uvaria (L.) Oken | Herb | Asphodelaceae | 11 | 10 | 9 |
| Koeleria macrantha (Ledeb.) Schult. | Graminoid | Poaceae | 6 | 6 | 6 |
| Lablab purpureus (L.) Sweet | Vine/scrambler/climber | Fabaceae | 31 | 30 | 28 |
| Lamarckia aurea (L.) Moench | Graminoid | Poaceae | 32 | 32 | 31 |
| Lathyrus angulatus L. | Herb | Fabaceae | 8 | 8 | 8 |
| Lathyrus latifolius L. | Herb | Fabaceae | 8 | 8 | 7 |
| Lathyrus nissolia L. | Herb | Fabaceae | 4 | 3 | 3 |
| Lathyrus odoratus L. | Herb, Vine/scrambler/climber | Fabaceae | 10 | 9 | 9 |
| Lepidium sativum L. | Herb | Brassicaceae | 14 | 13 | 12 |
| Lippia alba (Mill.) N.E.Br. ex Britton & P.Wilson | Shrub | Verbenaceae | 48 | 43 | 37 |
| Lonicera periclymenum (Unresolved) | Vine/scrambler/climber | Caprifoliaceae | 5 | 5 | 4 |
| Lycium barbarum L. | Shrub | Solanaceae | 7 | 6 | 5 |
| Macroptilium lathyroides (L.) Urb. | Herb | Fabaceae | 81 | 76 | 65 |
| Malva moschata L. | Herb | Malvaceae | 3 | 3 | 3 |
| Matthiola incana (L.) R.Br. | Herb | Brassicaceae | 13 | 14 | 14 |
| Mecardonia procumbens (Mill.) Small | Herb | Plantaginaceae | 15 | 18 | 19 |
| Medicago arborea L. | Shrub | Fabaceae | 40 | 40 | 43 |
| Medicago littoralis Loisel. | Herb | Fabaceae | 22 | 24 | 26 |
| Medicago sativa L. | Herb | Fabaceae | 27 | 25 | 23 |
| Merremia quinquefolia (L.) Hallier f. | Herb | Convolvulaceae | 12 | 13 | 13 |
| Merremia tuberosa (L.) Rendle | Vine/scrambler/climber | Convolvulaceae | 1 | 1 | 1 |
| Miscanthus sinensis Andersson | Graminoid | Poaceae | 8 | 7 | 7 |
| Myosotis sylvatica Ehrh. ex Hoffm. | Herb | Boraginaceae | 7 | 6 | 6 |
| Ononis spinosa L. | Shrub | Fabaceae | 8 | 7 | 7 |
| Ornithogalum umbellatum L. | Herb | Asparagaceae | 4 | 4 | 3 |
| Osteospermum ecklonis (DC.) Norl. | Shrub | Asteraceae | 14 | 12 | 10 |
| Panicum antidotale Retz. | Graminoid | Poaceae | 88 | 89 | 87 |
| Panicum bulbosum Kunth | Graminoid | Poaceae | 6 | 8 | 10 |
| Panicum capillare L. | Graminoid | Poaceae | 14 | 12 | 10 |
| Panicum coloratum L. | Graminoid, Herb | Poaceae | 60 | 57 | 53 |
| Panicum repens L. | Graminoid | Poaceae | 74 | 74 | 69 |
| Panicum schinzii Hack. | Graminoid, Herb | Poaceae | 33 | 29 | 25 |
| Paspalum conjugatum P.J.Bergius | Graminoid | Poaceae | 9 | 9 | 8 |
| Paspalum notatum FlŸggŽ | Graminoid | Poaceae | 35 | 36 | 35 |
| Paspalum paniculatum L. | Graminoid | Poaceae | 8 | 8 | 8 |
| Paspalum plicatulum Michx. | Graminoid | Poaceae | 21 | 20 | 19 |
| Paspalum urvillei Steud. | Graminoid | Poaceae | 29 | 24 | 19 |
| Passiflora caerulea L. | Vine/scrambler/climber | Passifloraceae | 52 | 48 | 43 |
| Pennisetum glaucum (L.) R.Br. | Graminoid | Poaceae | 71 | 75 | 76 |
| Pennisetum pedicellatum Trin. | Graminoid | Poaceae | 20 | 21 | 22 |
| Petasites fragrans (Vill.) C.Presl | Herb | Asteraceae | 2 | 2 | 1 |
| Phalaris coerulescens Desf. | Graminoid | Poaceae | 6 | 6 | 6 |
| Phormium tenax J.R.Forst. & G.Forst. | Herb | Hemerocallidaceae | 1 | 1 | 1 |
| Phytolacca americana L. | Herb | Phytolaccaceae | 21 | 17 | 12 |
| Pinus contorta Douglas ex Loudon | Shrub, Tree | Pinaceae | 4 | 4 | 4 |
| Pinus nigra J.F.Arnold | Tree | Pinaceae | 4 | 4 | 4 |
| Pinus palustris Mill. | Tree | Pinaceae | 3 | 2 | 1 |
| Pinus pinea L. | Tree | Pinaceae | 15 | 17 | 23 |
| Pinus ponderosa Douglas ex C.Lawson | Tree | Pinaceae | 3 | 3 | 3 |
| Pinus sabiniana Douglas | Tree | Pinaceae | 2 | 2 | 2 |
| Pinus sylvestris L. | Tree | Pinaceae | 4 | 3 | 3 |
| Pinus taeda L. | Tree | Pinaceae | 11 | 6 | 2 |
| Piptochaetium montevidense (Spreng.) Parodi | Graminoid | Poaceae | 30 | 31 | 32 |
| Poa bulbosa L. | Graminoid, Herb | Poaceae | 20 | 19 | 18 |
| Poa compressa L. | Graminoid | Poaceae | 4 | 4 | 4 |
| Poa pratensis L. | Graminoid | Poaceae | 10 | 8 | 7 |
| Poa trivialis L. | Graminoid | Poaceae | 8 | 8 | 7 |
| Polygonum bellardii All. | Herb | Polygonaceae | 24 | 21 | 19 |
| Polygonum patulum M.Bieb. | Herb | Polygonaceae | 18 | 18 | 18 |
| Populus tremula L. | Tree | Salicaceae | 3 | 3 | 3 |
| Praxelis clematidea (Griseb.) R.M.King & H.Rob. | Herb | Asteraceae | 21 | 18 | 15 |
| Prunus amygdalus Batsch | Tree | Rosaceae | 17 | 18 | 18 |
| Prunus armeniaca L. | Tree | Rosaceae | 27 | 26 | 25 |
| Prunus avium (L.) L. | Tree | Rosaceae | 4 | 4 | 4 |
| Prunus cerasifera Ehrh. | Shrub, Tree | Rosaceae | 8 | 8 | 7 |
| Prunus domestica L. | Tree | Rosaceae | 4 | 4 | 4 |
| Prunus laurocerasus L. | Tree | Rosaceae | 3 | 3 | 3 |
| Prunus persica (L.) Stokes | Tree | Rosaceae | 26 | 23 | 19 |
| Prunus serotina Ehrh. | Shrub, Tree | Rosaceae | 9 | 8 | 7 |
| Prunus spinosa L. | Tree | Rosaceae | 11 | 10 | 9 |
| Pycreus flavescens (L.) P. Beauv. ex Rchb. | Graminoid | Cyperaceae | 25 | 22 | 20 |
| Quercus ilex L. | Tree | Fagaceae | 9 | 8 | 8 |
| Quercus robur L. | Tree | Fagaceae | 4 | 4 | 4 |
| Reseda alba L. | Herb | Resedaceae | 16 | 16 | 16 |
| Retama monosperma (L.) Boiss. | Shrub | Fabaceae | 27 | 34 | 40 |
| Rostraria festucoides (Link) Romero Zarco | Graminoid | Poaceae | 16 | 15 | 15 |
| Rubus idaeus L. | Shrub | Rosaceae | 4 | 4 | 4 |
| Rubus laciniatus (Unresolved) | Shrub | Rosaceae | 5 | 5 | 4 |
| Schinus molle L. | Tree | Anacardiaceae | 53 | 50 | 48 |
| Senna didymobotrya (Fresen.) H.S.Irwin & Barneby | Herb, Shrub | Fabaceae | 27 | 28 | 28 |
| Senna multiglandulosa (Jacq.) H.S.Irwin & Barneby | Shrub, Tree | Fabaceae | 12 | 12 | 12 |
| Senna siamea (Lam.) H.S.Irwin & Barneby | Tree | Fabaceae | 9 | 9 | 10 |
| Setaria palmifolia (J.Koenig) Stapf | Graminoid | Poaceae | 19 | 20 | 19 |
| Setaria sphacelata (Schumach.) Stapf & C.E.Hubb. ex Moss | Graminoid | Poaceae | 41 | 38 | 33 |
| Solanum abutiloides (Griseb.) Bitter & Lillo | Shrub | Solanaceae | 9 | 8 | 8 |
| Solanum americanum Mill. | Herb | Solanaceae | 52 | 48 | 45 |
| Solanum asperolanatum Ruiz & Pav. | Herb | Solanaceae | 4 | 4 | 5 |
| Solanum capsicoides Mart. | Herb, Shrub | Solanaceae | 17 | 15 | 11 |
| Solanum dulcamara L. | Herb, Vine/scrambler/climber, Shrub | Solanaceae | 5 | 4 | 4 |
| Solanum furcatum Dunal | Herb | Solanaceae | 7 | 7 | 8 |
| Solanum laxum Spreng. | Vine/scrambler/climber | Solanaceae | 16 | 13 | 11 |
| Solanum lycopersicum Lam. | Herb | Solanaceae | 34 | 31 | 29 |
| Solanum melongena L. | Herb | Solanaceae | 47 | 47 | 47 |
| Solanum nigrescens M. Martens & Galeotti | Herb, Shrub | Solanaceae | 56 | 55 | 54 |
| Solanum orbiculatum (Unresolved) | Shrub | Solanaceae | 52 | 51 | 49 |
| Solanum pseudocapsicum L. | Shrub | Solanaceae | 49 | 44 | 39 |
| Solanum radicans L. f. | Herb | Solanaceae | 28 | 23 | 19 |
| Solanum sisymbriifolium Lam. | Herb | Solanaceae | 46 | 41 | 35 |
| Solanum tuberosum L. | Herb | Solanaceae | 7 | 6 | 6 |
| Solanum villosum Mill. | Herb | Solanaceae | 17 | 16 | 15 |
| Spergula pentandra L. | Herb | Caryophyllaceae | 15 | 14 | 13 |
| Stylosanthes humilis Kunth | Herb | Fabaceae | 24 | 25 | 25 |
| Stylosanthes viscosa Sw. | Herb | Fabaceae | 20 | 19 | 18 |
| Tarenaya hassleriana (Chodat) H.H. Iltis | Herb | Cleomaceae | 26 | 18 | 12 |
| Tephrosia nana Schweinf. | Herb | Fabaceae | 8 | 8 | 8 |
| Tradescantia zebrina Bosse | Herb | Commelinaceae | 16 | 14 | 13 |
| Tragopogon dubius Scop. | Herb | Asteraceae | 2 | 1 | 1 |
| Trifolium fragiferum L. | Herb | Fabaceae | 17 | 17 | 16 |
| Triumfetta bogotensis DC. | Shrub | Malvaceae | 8 | 8 | 7 |
| Verbascum blattaria L. | Herb | Scrophulariaceae | 8 | 7 | 6 |
| Viburnum tinus (Unresolved) | Shrub | Adoxaceae | 10 | 10 | 10 |
| Vicia sativa L. | Herb | Fabaceae | 24 | 23 | 22 |
| Vinca minor L. | Vine/scrambler/climber | Apocynaceae | 4 | 3 | 3 |
