# Supplementary material for: Next-Generation Invaders? Hotspots for Naturalised Sleeper Weeds in Australia under Future Climates
Source: PLoS One. 2013 Dec 26;8(12):e84222. doi: 10.1371/journal.pone.0084222 (PMC3873406; doi:10.1371/journal.pone.0084222)
Supplement: Table S2 — Global climate models used to simulate potential future climate scenarios across Australia. (DOCX) [file pone.0084222.s003.docx]

**Table S2: Global climate models used to simulate potential future climate scenarios across Australia**

| Abbreviation | Full title | Authors |
| --- | --- | --- |
| MRI-CGCM232A | Coupled Global Climate Model | Canadian Centre for Climate Modelling and Analysis |
| UKMO-HADGEM1 | Hadley Centre Global Environmental Model, Version 1 | Hadley Centre for Climate Prediction and Research, Met Office, United Kingdom |
| MPI-ECHAM5 | European Center Hamburg Version 5 | Max Planck Institute for Meteorology, Hamburg Germany |
| GFDL-CM20 | Geophysical Fluid Dynamics Laboratory Climate Model20 | Geophysical Fluid Dynamics Laboratory, US |
| UKMO-HADCM3 | United Kingdom Met Office – Hadley Centre Climate Model 3 | Hadley Centre for Climate Prediction and Research, Met Office, United Kingdom |
| CSIRO-MK30 | CSIRO Mk 3.0 | CSIRO Atmospheric Research, Australia |
| MIROC 3.2 | K-1 Coupled GCM (MIROC) version 3.2 medium resolution | Centre for Climate System Research, University of Tokyo; National Institute for Environmental Studies; Frontier Research Centre for Global Change |
